# Chronic obstructive sleep apnea promotes aortic remodeling in canines through miR-145/Smad3 signaling pathway

**DOI:** 10.18632/oncotarget.17144

**Published:** 2017-04-17

**Authors:** Chengyuan Yu, Yang Liu, Li Sun, Dingyu Wang, Yike Wang, Shiqi Zhao, Hui Dai, Jing Zhao, Song Zhang, Minghui Li, Yu Han, Shuang Lu, Xinwen Dong, Guangzhong Liu, Shengzhu Yu, Yue Li

**Affiliations:** ^1^ Department of Cardiology, The First Affiliated Hospital, Harbin Medical University, Harbin, 150001, Heilongjiang Province, P. R. China; ^2^ Key Laboratory of Cardiac Diseases and Heart Failure, Harbin Medical University, Harbin, 150001, Heilongjiang Province, P. R. China; ^3^ Institute of Metabolic Disease, Heilongjiang Academy of Medical Science, Harbin, 150086, Heilongjiang Province, P. R. China; ^4^ College of Resources and Environment, Northeast Agricultural University, Harbin, 150030, Heilongjiang Province, P. R. China

**Keywords:** obstructive sleep apnea, canine, aortic remodeling, miR-145, Smad3

## Abstract

Obstructive sleep apnea (OSA) is a causal pathogenetic factor of many cardiovascular diseases, however, its role in aortic diseases remains unknown. Therefore, this study was performed to explore the potential effects and pathophysiological mechanisms of chronic OSA on aortic remodeling in a canine model. After chronic OSA, the morphological changes of ascending aorta were characterized by thinner cells with pycnotic nuclei and swollen mitochondria, and obvious hyperplasia of collagenous fiber in the matrix. Both the apoptotic ratio and collagen volume fraction were significantly increased in ascending aorta of chronic OSA canines. Besides, aortic sympathetic nerve sprouting increased significantly in chronic OSA group. Meanwhile, protein expression of TGF-β1, Smad3, collagenI, apoptosis-inducing factor (AIF), tyrosine hydroxylase (TH) and growth associated protein-43 (GAP43) was upregulated after chronic OSA. Additionally, chronic OSA also strikingly increased pro-inflammatory factors like tumor necrosis factor α (TNF-α), NOD-like receptor 3 (NLRP3), NF-κB-p65 and oxidative stress factors like xanthine oxidase (XOD), malondialdehyde (MDA) while declined superoxide dismutase (SOD) activity. Furthermore, suppressed miR-145 and subsequently increased Smad3 expression were found obviously in vascular smooth muscle cells (VSMCs) treated by hypoxia. Luciferase reporter assays confirmed that Smad3 was one of the targets of miR-145. In conclusion, OSA could exacerbate aortic remodeling by aortic fibrosis, apoptosis and sympathetic nerve sprouting. miR-145/Smad3 signaling pathway might promote aortic remodeling during OSA. These findings provide novel information of chronic OSA-induced vascular dysfunction.

## INTRODUCTION

Obstructive sleep apnea (OSA) is a common sleep disorder that associated with high prevalence of 15% in men and 5% in women (aged 30-70 years) [1]. It is characterized by recurrent arousals, desaturations and hypercapnia resulting from upper airway collapse during sleep [2]. Accumulative evidence suggests that OSA is associated with increased cardiovascular morbidity and mortality, including hypertension, heart failure, coronary artery disease, and arrhythmia [3].

Recent studies have shown that OSA is confirmed to be one of the critical pathogenic casual factors in vascular dysfunction [4, 5]. OSA is highly prevalent in patients with aortic aneurysms and aortic dissection [6, 7]. It was demonstrated that OSA was an independent risk factor of aortic root size [8]. Severe OSA was also associated with greater extent of abdominal aortic calcification [9]. Tsioufis *et al*. [10] reported a significant incremental effect of OSA on aorta stiffening in hypertensive subjects. In addition, Kohler *et al*. [11] demonstrated a considerably higher prevalence of OSA in Marfan's syndrome patients, meanwhile, OSA promoted aortic dilatation and associated aortic dissection in patients with Marfan's syndrome. Despite these clinical findings, the concrete effects and mechanisms of chronic OSA in aortic diseases have not yet been clearly confirmed, mainly owing to many closely related confounders (*e.g*. age, sex, obesity, hypertension, *etc*.).

The present study aimed to clarify the pathologic changes of aortic structures and elucidate the underlying mechanism in a chronic OSA canine model.

## RESULTS

### Arterial blood gases and electrolytes analysis after acute apnea

To validate OSA model, arterial blood gases and electrolytes detection were performed after acute apnea for 60 s. Significantly decreased values of pH, pO_2_ and oxygen saturation O_2_ (SaO_2_), and increased levels of pCO_2_, HCO_3_^-^ and total CO_2_ (TCO_2_) were found after acute apnea (Table 1). At the same time, there was no differences of blood electronlytes between baseline and apnea.

**Table 1:** Arterial blood gases and electrolytes during acute apnea

	Baseline	Apnea
pH	7.40±0.02	7.29±0.03^*^
pCO_2_ (mmHg)	31.00±3.61	51.00±6.00^*^
pO_2_ (mmHg)	90.33±4.16	20.67±4.16^**^*
HCO_3_^-^ (mmol/L)	20.20±0.95	24.10±2.05*
TCO_2_ (mmol/L)	21.10±1.05	25.63±2.25*
SaO_2_ (%)	96.33±0.58	26.67±7.77^**^*
Na^+^ (mmol/L)	139.67±2.08	140.33±1.53
K^+^ (mmol/L)	3.73±0.15	3.67±0.67
Ca^2+^ (mmol/L)	1.28±0.08	1.31±0.07
BE (mmol/L)	-2.73±0.81	-2.63±1.22
BEecf (mmol/L)	-3.63±1.00	-2.73±1.05

SaO2 oxygen saturation, BE base excess, BEecf base excess of extracellular fluid.

*P < 0.05, ^*^P < 0.01, ^**^*P <0.001 vs. sham group, n=6 in each group.

### Morphology changes of ascending aorta smooth muscle in chronic OSA canines

**Figure 1:**
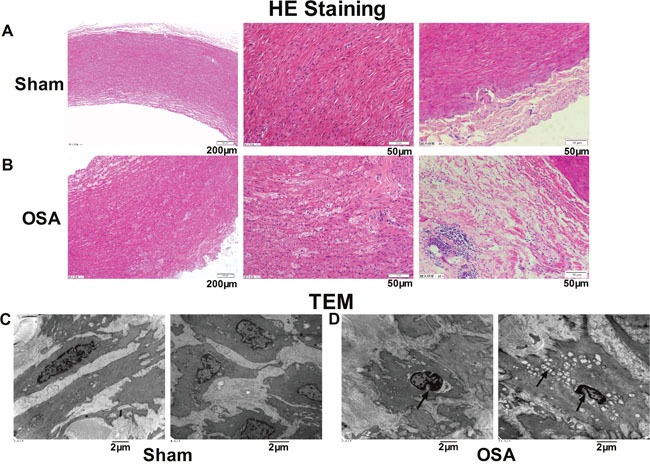
Morphological alterations in canine aorta after chronic OSA **(A, B)** Hematoxylin and eosin (HE) staining from ascending aorta of canines. Magnification: 100×, scale bar: 200 μm and magnification: 400×, scale bar: 50 μm. **(C, D)** Transmission electron microscope images showed the ultrastructural changes of ascending aorta in sham and OSA dogs. Magnification: 10000× and magnification: 12000×, scale bar: 2 μm.

Hematoxylin and eosin (HE) staining and transmission electron microscopy were conducted to examine the morphology alterations of ascending aorta smooth muscle cells. As shown in Figure 1A, normally and tightly arranged elastic lamella and aligned nuclei of the smooth muscle cells were observed in the sham group. However, OSA dogs revealed markedly increased aortic media and adventitia thickness, disordered and loosely arrangement or breakage of elastic fibers as well as the different orientated nuclei (Figure 1B). In addition, evident inflammatory cells infiltration were observed in the aortic adventitia of OSA dogs compared with sham dogs. As illustrated in Figure 1C, smooth muscle in sham dogs presented regular structure under transmission electron microscopy, whereas thinner cells with pycnotic nuclei and swollen mitochondria along with obvious hyperplasia of collagenous fibers in the matrix were observed in chronic OSA dogs (Figure 1D). Accordingly, these findings indicated that chronic OSA caused significant morphological changes of ascending aorta smooth muscle.

### Aortic interstitial fibrosis in chronic OSA canine model

**Figure 2:**
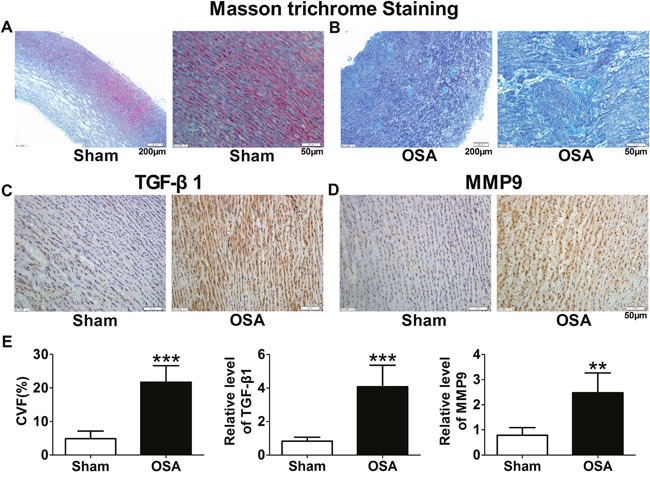
Fibrosis of aorta in sham and chronic OSA canines **(A, B)** Representative Masson trichrome staining images from ascending aorta in chronic OSA dogs. Magnification: 100×, scale bar: 200 μm and magnification: 400×, scale bar: 50 μm respectively. **(C)** Representative images of TGF-β1 expression in ascending aorta; **(D)** Representative images of MMP9 expression in ascending aorta. The magnification is 400×, scale bar: 50 μm. **(E)** Collagen volume fraction (CVF) and statistical results for expression of TGF-β1 and MMP9 in ascending aorta after chronic OSA. ^*^P < 0.01, ^**^*P <0.001 vs. sham group, n=6 in each group.

Based on the alterations of aortic morphological changes, we further observed fibrosis of ascending aorta in chronic OSA dogs by Masson staining. As shown in Figure 2A, chronic OSA group displayed a large amount of collagen fibers loosely and disorderly packed instead of the normal tissue (Figure 2B). Collagen volume fraction (CVF) was significantly increased in OSA dogs (*P* < 0.001, Figure 2E). Immunohistochemistry was performed to detect the protein expression of TGF-β1 and MMP9 in smooth muscle cells. While the exhibition of tan-color protein of TGF-β1, MMP9 increased dramatically in chronic OSA canines (*P* < 0.01, Figure 2C, 2D, 2E).

**Figure 3:**
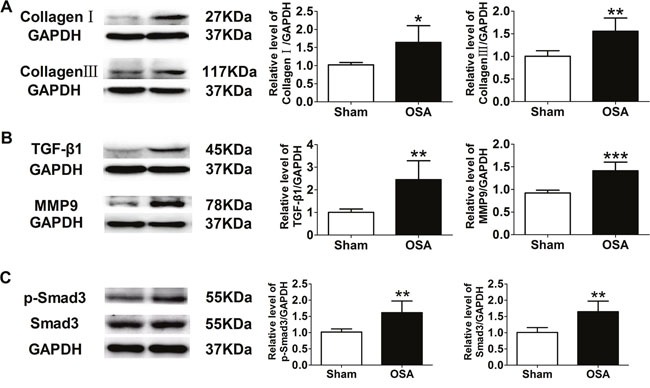
Expression of fibrosis-related proteins in sham and chronic OSA canines **(A)** Representative bands of CollagenI and III and ratio of these proteins to GAPDH. **(B)** Representative bands of TGF-β1 and MMP9, data from these proteins were normalized to GAPDH. **(C)** Representative bands of phospho-Smad3 and Smad3 as well as the ratio of these proteins to GAPDH. *P < 0.05, ^*^P < 0.01, ^**^*P <0.001 vs. sham group, n=6 in each group.

Protein expression of collagenI and III, TGF-β1 and MMP9 was upregulated in chronic OSA dogs (*P* < 0.05, Figure 3A, 3B), which was consistent with immunohistochemistry staining results (*P* < 0.01, Figure 2C, 2D). Importantly, as a key factor related to fibrosis the expression and activity of Smad3 were significantly increased in chronic OSA canines (*P* < 0.01, Figure 3C). Collectively, these data strongly supported that chronic OSA induced apparent aortic fibrosis.

### Aortic smooth muscle cells apoptosis in chronic OSA canines

**Figure 4:**
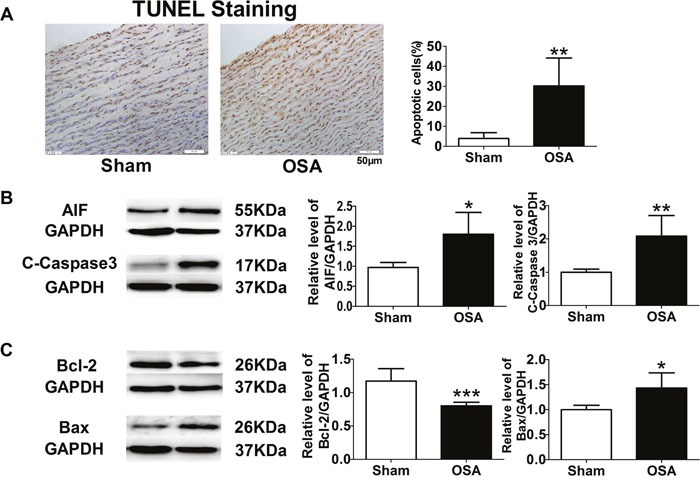
Apoptosis-related factors in sham and chronic OSA canines **(A)** TUNEL staining, magnification: 400×, scale bar: 50 μm, and ratio of TUNEL positive cells to total cells. **(B, C)** Representative bands of AIF, Cleaved-Caspase 3, Bcl-2 and Bax, data from these proteins were normalized to GAPDH.*P < 0.05, ^*^P < 0.01, ***P < 0.001 vs. sham group, n=6 in each group.

Besides aortic obvious interstitial fibrosis, TUNEL staining was employed for apoptotic cell detection. More brown-stained nuclei standing for apoptotic cells were found in OSA dogs as shown in Figure 4A (*P* < 0.01). We further examined expression of apoptosis-related proteins, such as apoptosis-inducing factor (AIF), Caspase 3, B cell lymphoma/lewkmia-2 (Bcl-2) and Bcl-2 Associated X Protein (Bax) and found that chronic OSA increased protein levels of AIF, Caspase 3 and Bax, while decreased protein level of Bcl-2 compared with sham group (*P* < 0.05, Figure 4B, 4C).

### Autonomic nerve remodeling in chronic OSA canines

**Figure 5:**
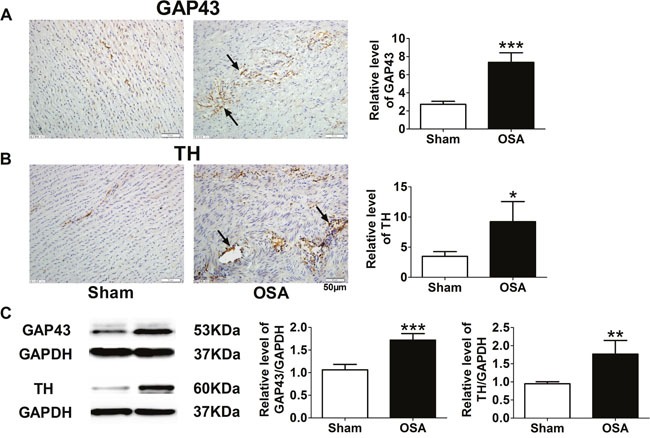
Sympathetic nerve sprouting and innervation of nerve fibers in ascending aorta of sham and chronic OSA dogs **(A)** Representative images and statistical results for expression of growth-associated protein 43 (GAP-43) in ascending aorta. **(B)** Representative images and statistical results for tyrosine hydroxylase (TH) expression in ascending aorta. Magnification: 400×, scale bar: 50 μm. **(C)** Representative bands of GAP43 and TH as well as the ratio of these proteins to GAPDH. *P < 0.05, ^*^P < 0.01, ^**^*P <0.001 vs. sham group, n=6 in each group.

In previous studies, we observed chronic OSA induced autonomic nerve remodeling in atria and ventricles. Therefore, we further detected whether autonomic nerve remodeling happened in ascending aorta. We found that endogenous neuronal growth-associated protein (GAP43) and tyrosine hydroxylase (TH), as markers for sympathetic nerve, increased markedly in chronic OSA dogs compared with sham group (Figure 5A, 5B). In addition, nerve fibres were enlarged and innervated in disorder. Consistently, expressions of GAP43 and TH were also upregulated after chronic OSA (*P* < 0.01, Figure 5C). Thus, it suggested that chronic OSA led to sympathetic sprouting and sympathetic hyperinnervation in ascending aorta.

### Inflammation and oxidative stress in chronic OSA model

**Figure 6:**
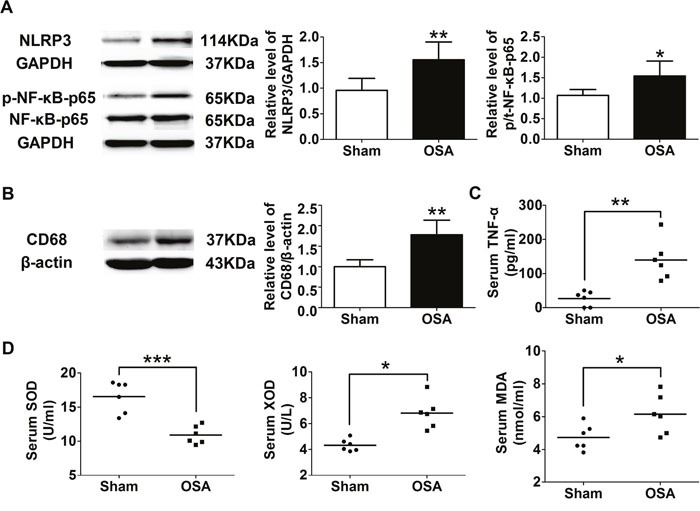
Inflammation and oxidative stress associated factors of sham and chronic OSA dogs **(A)** Representative bands of NLRP3, phospho-NF-κB-p65 and NF-κB-p65 as well as the ratio of NLRP3 to GAPDH and phospho-NF-κB-p65 to NF-κB-p65. **(B)** Representative band of CD68 and the ratio of CD68 to β-actin. **(C, D)** Serum levels of TNF-α, SOD activity, XOD and MDA. *P < 0.05, ^*^P < 0.01, ^**^*P <0.001 vs. sham group, n=6 in each group.

Next, we tried to explore the role of inflammation and oxidative stress in aortic remodeling above after chronic OSA. Compared with the sham group, the inflammation associated protein level of NOD-like receptor 3 (NLRP3), phospho-NF-κB p65 and CD68, a specific marker for macrophage, was markedly higher in chronic OSA dogs (*P* < 0.05, Figure 6A, 6B). The serum level of *tumor* necrosis factor α (TNF-α), superoxide dismutase (SOD), xanthine oxidase (XOD), and malondialdehyde (MDA) was also employed by ELISA assays. Serum level of pro-inflammation cytokine TNF-α in the OSA canines showed an increase trend (*P* < 0.01, Figure 6C). Besides, activity of oxidative stress related factor SOD was observed markedly decreased while XOD and MDA were notably increased in OSA group (*P* < 0.05, Figure 6D). These data suggested that inflammation and oxidative stress were involved in aortic remodeling of chronic OSA.

### Systemic blood pressure in acute OSA canines

Firstly, the femoral and pulmonary pressures at baseline and after acute OSA were measured. Both the systolic pressure and mean pressure of femoral and pulmonary artery elevated after acute apnea for both 60 and 90 s (Supplementary Figure 1A, 1B). Systolic arterial pressure (SAP), pulmonary artery systolic pressure (PASP) and pulmonary artery mean pressure (PAMP) were significantly increased after apnea for 90 s (*P* < 0.05). In contrast, acute apnea did not significantly change diastolic arterial pressure (DAP), mean arterial pressure (MAP) and pulmonary artery diastolic pressure (PADP) (*P* > 0.05).

### MiR-145/Smad3 signaling mainly promotes aortic remodeling in chronic OSA

**Figure 7:**
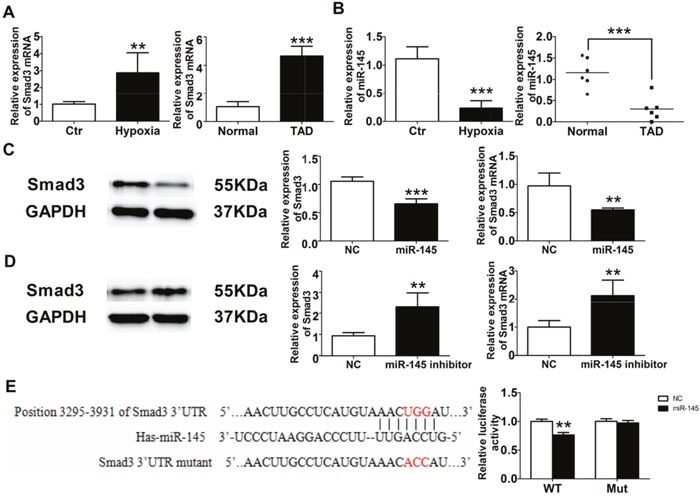
miR-145/Smad3 signal pathway promotes aortic remodeling **(A)** Smad3 expression from VSMCs and TAD patients. **(B)** miR-145 expression from VSMCs and TAD patients. Western blot and RT-PCR detection of Smad3 expression from VSMCs after miR-145 mimics **(C)** and miR-145 inhibitor **(D)**. **(E)** Dual luciferase reporter assay of WT and Mut Smad 3’ UTR.*P < 0.05, ^*^P < 0.01, ^**^*P <0.001 vs. Control or Normal group, n=6 in each group.

Smad3 was significantly increased in vascular smooth muscle cells (VSMCs) treated by hypoxia and aortic tissue of thoracic aortic dissection (TAD) patients (Figure 7A), which was consistent with that of OSA canines. The expression of miR-145 was notably decreased in VSMCs treated by hypoxia and TAD patients (Figure 7B). In order to access whether Smad3 was targeted by miR-145, VSMCs were transfected with miR-145 mimics or inhibitor. As shown in Figure 7C, 7D, Smad3 expression was downregulated by miR-145 mimics and upregulated by miR-145 inhibitor. Furthermore, we performed a luciferase assay using a vector carrying wild-type (WT) or mutant (Mut) Smad3 3’ –UTR and co-transfected them with miR-145 mimics or negative control (NC). Luciferase reporter assays showed that miR-145 significantly suppressed the luciferase activities in HEK-293T cells transfected with the Smad3 3’-UTR-WT reporter, whereas they did not have any significant influence on the luciferase activities of the mutant reporter (Figure 7E), suggesting that Smad3 was targeted by miR-145 directly.

## DISCUSSION

In our present study, we demonstrated that chronic OSA could cause aortic structural and autonomic nerve remodeling by promoting aortic fibrosis, apoptosis and sympathetic nerve sprouting and innervating in a canine model. It was mainly promoted by miR-145/Smad3 signaling pathway.

Increasing evidence suggests that OSA is a causal factor in the pathogenesis of vascular dysfunction and hypertension, which can promote dilation and subsequent dissection [4]. Our present study found obvious incrassation of media and adventitia thickness, elastic fibers disorganization and hyperplasia of collagenous fiber in OSA dogs (Figure 1A, 1B), which might be implicated in early phase of atherosclerosis [12]. To explore the mechanism, MMP9, CollagenI and III were found increased (Figure 3A, 3C), suggesting degradation of extracellular matrix and collagen deposition [13]. Furthermore, as the downstream signal transducer of TGF-β1 [14], total and phosphorylation of Smad3 were significantly upregulated (Figure 3C).

In this study, we observed femoral arterial pressure and pulmonary arterial pressure were elevated after apnea for 60 s and 90 s compared with baseline (Supplementary Figure 1A, 1B), indicating that acute apnea increased blood pressure. According to our previous study, negative intrathoracic pressure was markedly enhanced after apnea for 30 s and 60 s [15]. The intrathoracic pressure swinged to stretch the aortic wall and led to increased transmural gradients, which generated enhanced pathological shear stress on the aorta together with the blood pressure rose. These changes might cause impairment of aortic elasticity property (*e.g*. fibrin fragmentation, collagen deposition, *etc*.), ultimately resulted in aortic dilatation and subsequently dissection [4–6, 16].

During OSA, cessation of airway leads to intermittent hypoxia and negative intrathoracic pressure swings accompanied by recurrent arousals, which consequently accelerates the elevation of blood pressure and sympathetic activation [17]. Our previous study reported that chronic OSA caused atrial sympathetic hyperinnervation in canines [15]. Furthermore, in this study, we observed increased protein expression of TH and GAP43 (Figure 5) in OSA dogs, indicating sympathetic nerve sprouting and innervating disorderly in the aorta after OSA stimulation.

In the present study, we demonstrated the OSA induced aortic inflammation, as shown by increased level of serum TNF-*α* and expression of NLRP3, phospho-NF-κB p65 and CD68 in the aorta after 12 weeks of OSA, which was accompanied with increased expressions of markers of oxidative stress (XOD, MDA), cell death (cleaved caspase3, AIF and BAX) and remodeling (aorta tunica media thickness, collagen accumulation, and the expression of TGF-*β*1) in OSA group. These findings are consistent with the previous concept that inflammation and oxidative stress are reciprocal causes and outcomes, both of which are main pathogenic factors for the development of aortic remodeling under OSA [5]. The effects of chronic inflammation including induction of oxidative stress, apoptotic cell death, all of which could contribute to the structural and functional abnormalities of the cells, which subsequently contribute to the derangement of aortic structure [5]. In turn, excessive ROS caused by OSA could increase expression of adhesion molecules and inflammatory cytokines, which promotes endothelial dysfunction and is the prelude to atherosclerosis [18]. Take together, firstly, intermittent hypoxia evokes severe systemic inflammatory and oxidative stress response, then interacts with each other, which leads to the subsequent aortic remodeling in OSA canine model.

Several studies showed that Smad3 was critical for macrophage/monocyte chemotaxis and thus evoked inflammatory reaction [19], besides Smad3 could increase ROS production [20]. What is more, Smad3 can accelerate vascular fibrosis and collagen deposition [21, 22]. We found that the expression and activity of Smad3 were activated significantly in aorta tissue of chronic OSA canines (Figure 3C). Which indicated that Smad3 might play a great role in aorta remodeling in a chronic OSA canine model. In addition, hypoxia induced obviously increased expression of Smad3 and p-Smad3 on VSMCs (Supplementary Figure 2B). Accumulating evidence that implicates miRNAs’ involvement in a variety of biological processes in diseased vessels. We then searched for the upstream miRNA of Smad3 through TargetScan and miRbase and found miR-145 could target Smad3. Whereafter, we confirmed that Smad3 was one of the targets of miR-145 by Luciferase reporter assays (Figure 7E). We further validated that repressed miR-145 and increased Smad3 expression were observed on thoracic aortic dissection (TAD) patients (Figure 7A, 7B), which suggested that miR-145/Smad3 signaling pathway involved in aorta remodeling on thoracic aortic dissection TAD patients.

In conclusion, this study demonstrated that the OSA-induced aortic remodeling attributed to the process of fibrosis, apoptosis and sympathetic nerve sprouting, which might be initiated by inflammation and oxidative stress promoted by miR-145/Smad3 signaling pathway. Our findings offered new perspectives to discover the pathogenesis of OSA and provided novel strategy for treatment of OSA-induced aortic remodeling.

## MATERIALS AND METHODS

### Animals

All animal care and handling procedures were approved by the ethic committees of Harbin Medical University, and the whole experiments in this study were complied with the Guide for the Care and Use of Laboratory Animals published by the US National Institutes of Health (8th edition, 2011). Twelve healthy mongrel dogs (male, weight 15~20 kg) were provided by Experimental Animal Center of the First Affiliated Hospital of Harbin Medical University (Harbin, China). Dogs were anesthetized with ketamine (5.3 mg/kg, iv), diazepam (0.25 mg/kg, iv), and xylazine (1 mg/kg, iv). Adequacy of anesthesia was monitored according to the disappearance of the corneal reflex and jaw tone. Dogs were randomly divided into sham (anesthesia and tracheal intubation only) and chronic OSA group.

### OSA stimulation

The protocol of chronic OSA was followed by our previous studies [15, 23, 24]. Briefly, it was established by stopping the ventilator and closing the airway for 4 h apnea-ventilation cycles every other day for 12 weeks. Initially, the trachea blockage was 1 min and trachea ventilation was 9 min, therefore, the apnea hypopnea index was maintained 6. Then the duration of ventilation was shorter than the previous week gradually. The duration of trachea ventilation was 5 min and apnea hypopnea index was 10, which lasted for 8 weeks.

### Arterial blood gas and electrolytes analysis

Arterial blood was collected from femoral artery for measurement of pH, pCO_2_, pO_2_, HCO_3_^-^, TCO_2_ and SaO_2_ at baseline and acute apnea. Furthermore, electrolytes including Na^+^, K^+^, Ca^2+^, base excess (BE) and base excess of extracellular fluid (BEecf) were also determined [15].

### Transmission electron microscopy

A transmission electron microscopy (JEM-1220, JEOL Ltd., Tokyo, Japan) was used to examine the ultrastructures of ascending aorta smooth muscle tissues. Samples were fixed in 2.5% glutaraldehyde in 0.1mol/L PBS (PH 7.35) and followed by 1% osmium tetroxide, stained in 1% uranyl acetate, dehydrated in ethanol, and embedded in epoxy resin as described in our previous study [25].

### HE and masson trichrome staining

Ascending aortic samples were fixed for 24 h in 10% neutral buffered formalin and paraffin embedded, then cut into 5 μm slices. After that, they were stained with HE or Masson trichrome for histological and collagen analysis. The interstitial fibrosis areas were calculated by image analysis software (Image-pro plus 6.0, Meida Cybernetics LP), CVF was calculated as collagen area/total area×100% [26].

### Immunohistochemistry

Immunohistochemistry was performed on paraffin-embedded ascending aorta sections. Slices were incubated with anti-TGF-β1 (ab92486, Abcam, USA), anti-MMP9 (bs-4593R, Bioss, China), anti-GAP43 (ab12274, Abcam, USA), and anti-TH (ab112, Abcam, USA) overnight at 4°C. The slides were reacted with peroxidase conjugated goat anti-rabbit IgG (ZDR-5306, Zhongshan, China) at 37°C for 20 min [15, 27].

### TUNEL staining

Formalin-fixed, paraffin-embedded sections were stained by the TUNEL assay according to manufacturer's instructions (Roche, Indianapolis, IN, USA). The smooth muscle cells of ascending aorta, which were stained in brown nuclei, were positive under an Olympus BX-60 microscope (Olympus, Tokyo, Japan). Apoptotic rate was calculated as the ratio of positively stained cells/total smooth muscle cells [26].

### Enzyme-linked immunosorbent assay

Enzyme-linked immunosorbent assay (ELISA) was used to detect protein expression of serum TNF-α (E03T0008, Blue Gene Biotech, China), SOD, XOD and MDA (A002, A001-3, A003-1, Nanjing Jiancheng Bioengineering Institute, China) according to the instructions.

### Western blotting

Total protein samples were extracted from ascending aorta according to described previously [15, 26]. Protein were separated on 10% sodium dodecyl sulfate-polyacrylamide gel electrophoresis and transferred to polyvinylidene fluoride membrane (Millipore, Bedford, MA, USA). After blocking with 5% nonfat dry milk, the membranes were then incubated with anti-CollagenI (bs-0578R, Bioss, China), anti-Collagen III (bs-0549R, Bioss, China), anti-TGFβ1 (ab92486, Abcam, USA), anti-α-SMA (ab5694, Abcam, USA), anti-MMP9 (bs-4593R, Bioss, China), anti-Smad3 (#9523, Cell Signaling Technology, USA), p-Smad3 (#9520, Cell Signaling Technology, USA), anti-AIF (ab1998, Abcam, USA), anti-cleaved caspase 3 (#9664, Cell Signaling Technology, USA), anti-caspase 9 (#9508, Cell Signaling Technology, USA), anti-Bcl-2 (ab32124, Abcam, USA), anti-Bax (ab32503, Abcam, USA), anti-GAP43 (ab12274, Abcam, USA), anti-TH (ab112, Abcam, USA), anti-NLRP3 (bs-10021R, Bioss, China), anti-NF-κB p65 (10745-1-AP, Proteintech, USA), anti-p-NF-κB p65 (#3033, Cell Signaling Technology, USA), anti-CD68 (ab76308, Abcam, USA), HIF-1α (R12-2180, Assaybiotech, USA), anti-GAPDH (KC-5G4, Kangchen, China) and anti-β-actin (TA-09, Zhongshan, China) antibodies in 4°C overnight. Then horseradish peroxidase-conjugated secondary antibody (ZB-2301, Zhongshan, China) was incubated for 1 h at 37°C. The images were obtained by exposure after reactions with enhanced chemiluminescence reagents, and were analyzed using Quantity One software (Bio-Rad, Hercules, CA, USA).

### Tissue samples collection

Ascending aorta segments were collected from thoracic aortic dissection (TAD) patients undergoing surgical repair and patients who underwent coronary artery bypass grafting surgery without aortic diseases (Normal) from the first affiliated hospital of Harbin Medical University. This study was approved by the ethics committee of Harbin Medical University. Six pair of normal and dissected aortic tissue were used for RNA extraction.

### Cell culture

Male Sprague Dawley rats were killed by cervical dislocation. Vascular smooth muscle cells (VSMCs) were prepared as previously described [28] from thoracic aortas and were cultured under a humidified air atmosphere containing 5% O_2_ at 37°C in Dulbecco's Modified Eagle's Medium (C11995500BT, Gibco, USA) containing 10% fetal bovine serum, penicillin (100mg/mL) and streptomycin (100U/mL). VSMCs were used between passage 3 and 8 in the experiments. In order to expose to hypoxia, cells were maintained in a hypoxia chamber (1% O_2_, 5% CO_2_; balance N_2_ and water vapor) for 24 h.

### MicroRNA transfection

MiR-145 mimic, miR-145 inhibitor and control RNA (Gene-Pharma, China) were transfected into VSMCs using Lipofectamine 2000 (Invitrogen, USA) according to the manufacturer's instructions. 48 h later, VSMCs were used for our experiments [29].

### RNA extraction and quantitative real-time PCR

Total RNA was extracted from tissues or cultured cells using the trizol reagent (Invitrogen, USA) according to manufacturer's instructions. Total RNA samples were reversely transcribed in cDNA using the miRcute reverse transcription kit (KR211, Tiangen, China) and a Prime Script™ RT reagent kit (Takara, Japan) following the instructions from the respective manufacturers. Real-time PCR was performed using a standard SYBR Green PCR kit (Toyobo, Japan) and ABI 7500 real-time PCR System (Applied Biosystems, USA). Each sample was assessed in triplicate. All fold changes were calculated by the method of 2^−ΔΔCt^ [30].

### Plasmid construction and luciferase reporter assay

Wild-type or mutant of 3’ untranslated region sequences of Smad3 were inserted into the BglII and XhoI sites of the pGL4.23 vector (Cat#E8411, Promega). Reporter plasmids containing 3'UTR Smad3 (pGL4-Smad3-UTR and pGL4-Smad3-UTR-mut) were co-transfected with miR-145 mimic NC or miR-145 mimic into HEK-293T cells. pRL-TK Renilla luciferase reporter vector was used as an internal control in each assay. Firefly and Renilla luciferase activities were measured using the Dual-Luciferase Reporter Assay system [31]. Results were expressed as firefly luciferase activity normalized to Renilla luciferase activity.

### Statistical analysis

All experimental data were expressed as mean ± SD. Comparison between two groups was conducted by Students’ test. *P* < 0.05 was considered statistically significant.

## SUPPLEMENTARY MATERIALS FIGURES



## Abbreviations

OSA: obstructive sleep apnea
HE: hematoxylin and eosin
AIF: apoptosis-inducing factor
C-Caspase 3: Cleaved-Caspase 3
C-Caspase 9: Cleaved-Caspase 9
Bcl-2: B cell lymphoma/lewkmia-2
Bax: Bcl-2 Associated X Protein
TH: tyrosine hydroxylase
GAP43: growth associated protein-43
TNF-α: *tumor* necrosis factor α
NLRP3: NOD-like receptor3
XOD: xanthine oxidase
MDA: malondialdehyde
SOD: superoxide dismutase
SAP: systolic arterial pressure
DAP: diastolic arterial pressure
MAP: mean arterial pressure
PASP: pulmonary artery systolic pressure
PADP: pulmonary artery diastolic pressure
PAMP: pulmonary artery mean pressure.
