# Supplementary material for: Chronic obstructive sleep apnea promotes aortic remodeling in canines through miR-145/Smad3 signaling pathway
Source: Oncotarget. 2017 Apr 17;8(23):37705–16. doi: 10.18632/oncotarget.17144 (PMC5514942; doi:10.18632/oncotarget.17144)
Supplement: Supplementary file 1 [file oncotarget-08-37705-s001.pdf]

Chronic obstructive sleep apnea promotes aortic remodeling in canines through miR-145/Smad3 signaling pathway

SUPPLEMENTARY DATA

Measurement of femoral and pulmonary artery pressures

Femoral and pulmonary artery pressures were measured after acute apnea for 60 s or 90 s according to previous studies [1]. Briefly, the 6F sheath was percutaneously induced into femoral artery to gauge the systolic arterial pressure (SAP), diastolic arterial pressure (DAP) and mean arterial pressure (MAP), while the sheath was inserted through femoral vein and advanced into the pulmonary artery to measure the pulmonary artery systolic pressure (PASP), pulmonary artery diastolic pressure (PADP) and pulmonary artery mean pressure (PAMP).

Zymography

MMP-activity was investigated using gelatinase zymography as previously described [2]. Briefly, canine samples were loaded on 10 % SDS-polyacrylamide gels containing gelatin at room temperature for 1 h. After electrophoresis, the gels were treated with Triton X-100 for 30 minutes at room temperature and incubated in a reaction buffer containing 50 mM Tris (pH 7.4), 5 mM CaCl2, and 150 mM NaCl for 24 hours at 37°C, then stained with Coomassie blue. Clear bands in the zymogram indicated enzymatic digestion of gelatin.

REFERENCES

1. Hori Y, Kondo C, Matsui M, Yamagishi M, Okano S, Chikazawa S, Kanai K, Hoshi F, Itoh N. Effect of the phosphodiesterase type 5 inhibitor tadalafil on pulmonary hemodynamics in a canine model of pulmonary hypertension. Veterinary journal. 2014; 202:334-339.
2. Gong L, Wu D, Zou J, Chen J, Chen L, Chen Y, Ni C, Yuan H. Prognostic impact of serum and tissue MMP-9 in non-small cell lung cancer: a systematic review and meta-analysis. Oncotarget. 2016; 7:18458-18468. doi: 10.18632/oncotarget.7607.

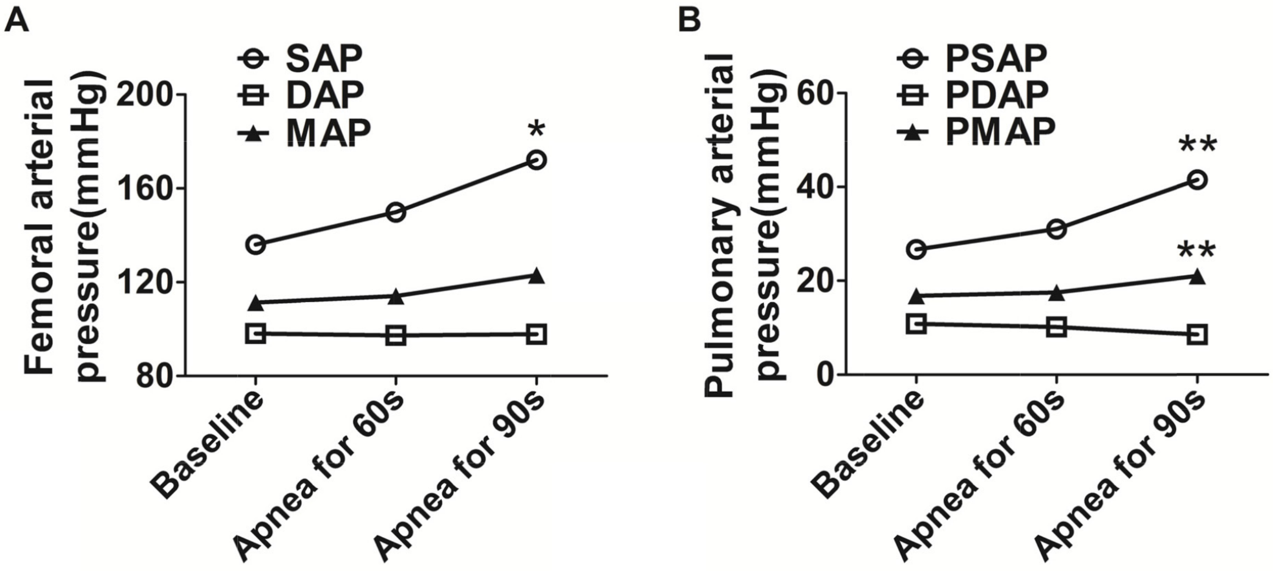

Supplementary Figure 1: Femoral and pulmonary artery pressures of sham and chronic OSA canines. (A) Representative femoral artery pressures. Systolic arterial pressure (SAP), diastolic arterial pressure (DAP), mean arterial pressure (MAP). **(B)** Representative pulmonary artery pressures. Pulmonary artery systolic pressure (PASP), pulmonary artery diastolic pressure (PADP), pulmonary artery mean pressure (PAMP), $^{*}P < 0.05$, $^{**}P < 0.01$ vs. baseline, n=6 in each group.

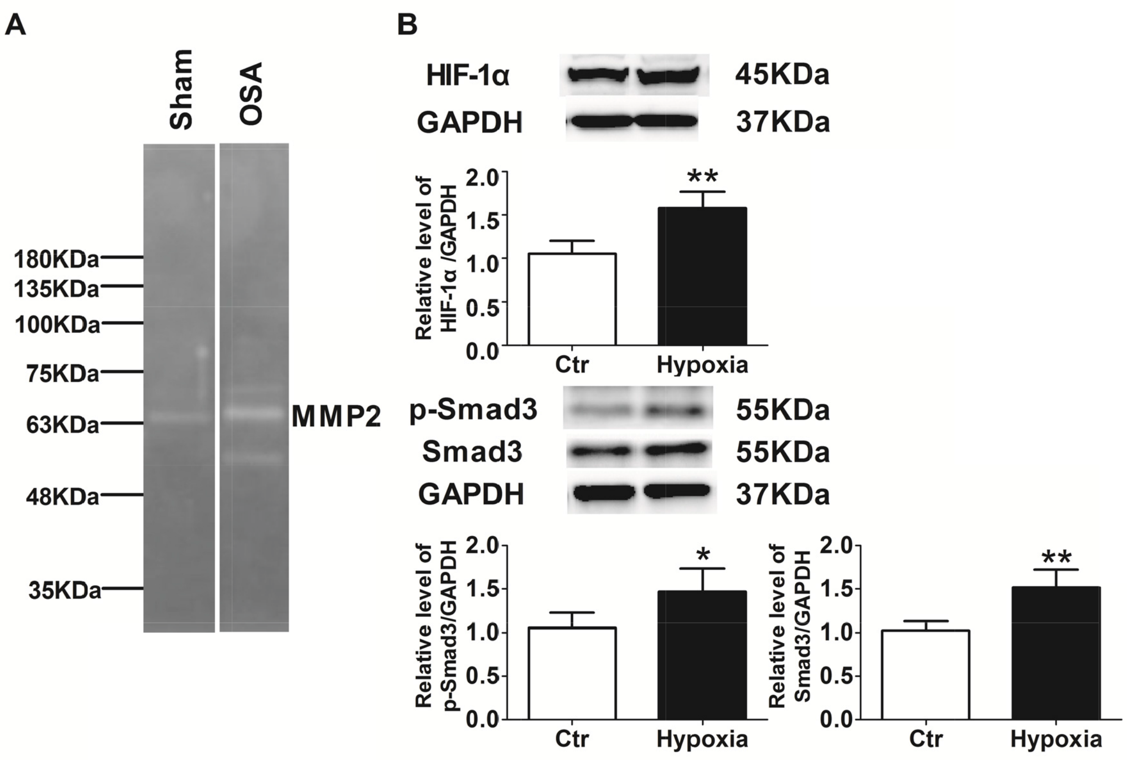

Supplementary Figure 2: Zymography and western blot of OSA canines and VSMCs. **(A)** Zymography of ascending aortic tissue from sham and OSA canines. **(B)** Protein expression of HIF-1α, p-Smad3 and Smad3 and relative ratio of these proteins to GAPDH from hypoxic treatment VSMCs by western blot. $^{*}P < 0.05$, $^{**}P < 0.01$ vs. control group, n=5 in each group.

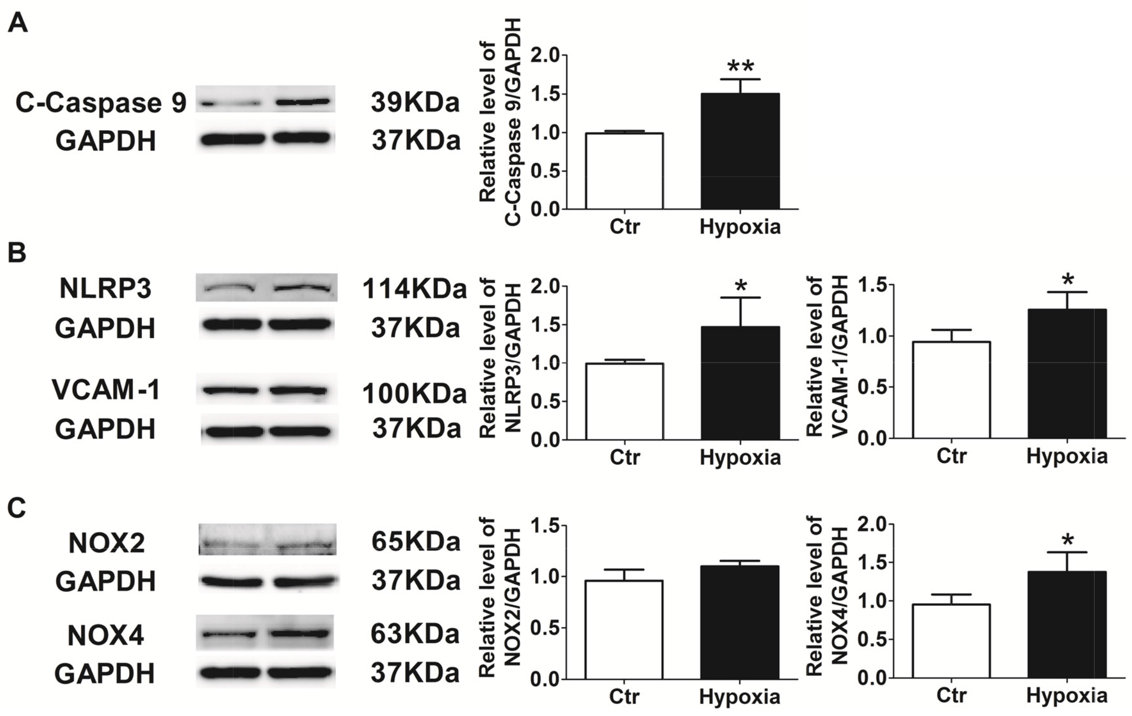

Supplementary Figure 3: Apoptosis, inflammation and oxidative stress-related factors in hypoxia treatment endothelial cells. **(A)** Protein expression of cleaved-caspase 9 in control and hypoxia treatment endothelial cells. **(B)** Representative bands of NLRP3 and VCAM-1, data from these proteins were normalized to GAPDH. **(C)** Protein expression of NOX2 and NOX4, and relative ratio of these proteins to GAPDH from hypoxic treatment endothelial cells by western blot. $^{*}P < 0.05$, $^{**}P < 0.01$ vs. control group, n=5 in each group.

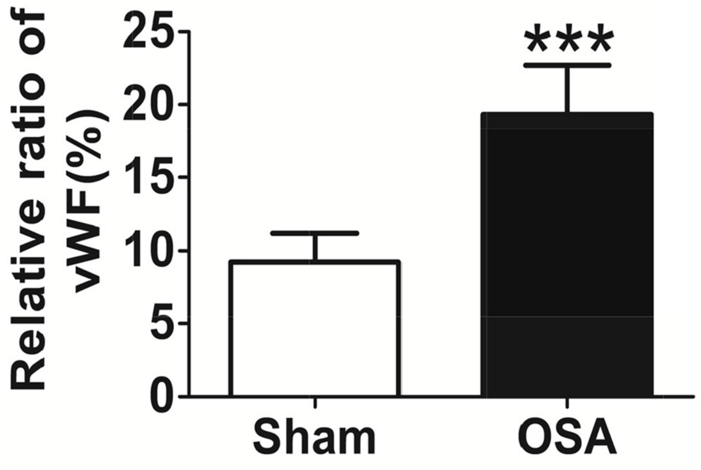

Supplementary Figure 4: Relative ratio of serum vWF (%) in sham and chronic OSA canines. $^{***}P < 0.001$ vs. sham group, n=5 in each group.
